# Nutraceutical Supplementation Ameliorates Visual Function, Retinal Degeneration, and Redox Status in rd10 Mice

**DOI:** 10.3390/antiox10071033

**Published:** 2021-06-26

**Authors:** Lorena Olivares-González, Sheyla Velasco, Isabel Campillo, David Salom, Emilio González-García, José Miguel Soriano del Castillo, Regina Rodrigo

**Affiliations:** 1 Pathophysiology and Therapies for Vision Disorders, Principe Felipe Research Center (CIPF), Eduardo Primo Yúfera 3, 46012 Valencia, Spain; lolivares@cipf.es (L.O.-G.); svelasco@cipf.es (S.V.); icampillo@cipf.es (I.C.); 2 Joint Research Unit on Rare Diseases CIPF-Health Research Institute Hospital La Fe (IIS-La Fe), 46012 Valencia, Spain; 3 Department of Ophthalmology, Manises Hospital, Av. de la Generalitat Valenciana, 50, 46940 Manises, Spain; dsalom@hospitalmanises.es (D.S.); egonzalezg@hospitalmanises.es (E.G.-G.); 4 CIBER de Enfermedades Raras (CIBERER), 28029 Madrid, Spain; 5 Food & Health Laboratory, Institute of Materials Science, University of Valencia (UV), Catedràtic José Beltrán Martínez, 2, 46980 Valencia, Spain; jose.soriano@uv.es; 6 Joint Research Unit on Endocrinology, Nutrition and Clinical Dietetics UV-IIS La Fe, 46026 Valencia, Spain; 7 Department of Physiology, University of Valencia (UV), Avda. Vicente Andrés Estellés, 50, Burjassot, 46100 Valencia, Spain

**Keywords:** retinitis pigmentosa, redox status, nutraceuticals, inflammation

## Abstract

Retinitis pigmentosa (RP) is a group of inherited retinal dystrophies characterized by progressive degeneration of photoreceptor cells. Ocular redox status is altered in RP suggesting oxidative stress could contribute to their progression. In this study, we investigated the effect of a mixture of nutraceuticals with antioxidant properties (NUT) on retinal degeneration in rd10 mice, a model of RP. NUT was orally administered to rd10 mice from postnatal day (PD) 9 to PD18. At PD18 retinal function and morphology were examined by electroretinography (ERG) and histology including TUNEL assay, immunolabeling of microglia, Müller cells, and poly ADP ribose polymers. Retinal redox status was determined by measuring the activity of antioxidant enzymes and some oxidative stress markers. Gene expression of the cytokines IL-6, TNFα, and IL-1β was assessed by real-time PCR. NUT treatment delayed the loss of photoreceptors in rd10 mice partially preserving their electrical responses to light stimuli. Moreover, it ameliorated redox status and reduced inflammation including microglia activation, upregulation of cytokines, reactive gliosis, and PARP overactivation. NUT ameliorated retinal functionality and morphology at early stages of RP in rd10 mice. This formulation could be useful as a neuroprotective approach for patients with RP in the future.

## 1. Introduction

Retinitis pigmentosa (RP) is a group of inherited retinal disorders characterized by progressive degeneration of photoreceptors (rods and cones), which eventually leads to blindness. It is a rare disease with a prevalence of approximately 1 in 4.000 individuals [1]. RP presents a high genetic and clinical heterogeneity, which makes it difficult to find the specific mutation, as well as the correct treatment. Patients with RP usually lose night vision and peripheral vision in the early stages of the disease, and eventually, central vision. The vision loss is mainly due to a gradual deterioration of photoreceptor cells, namely, rod degeneration, which is responsible for night and peripheral vision, followed by cone degeneration responsible for central and color vision. Genetic mutations affect rod survival but eventually promote cone degeneration. In particular, evidence indicates that rod degeneration triggers the release and accumulation of free radicals, inflammatory molecules, etc. that eventually would affect cone survival [2].

Oxidative stress is the consequence of an imbalance between the production and the accumulation of oxygen or nitrogen reactive species (ROS, RNS) and their elimination through endogenous antioxidant machinery in cells and tissues [3,4]. The retina is highly susceptible to oxidative damage by ROS. It is one of the highest oxygen-consuming tissues in the body [5]. Moreover, the retina is also predisposed to photo-oxidation due to its constant exposure to light. During rod degeneration, the degenerative retina is exposed to higher levels of O_2_, which results in ROS generation and contributes to cell death of the remaining rods and cones [2].

There is several lines of evidence of oxidative damage including ROS generation, oxidation of macromolecules such as lipids (malondialdehyde, MDA), proteins (protein carbonyls, CAR) and nucleic acids (8-oxoguanine, 8-oxo-dG), or downregulation of antioxidant effectors (antioxidant enzymes) in retinas of animal models of RP [6,7,8,9,10,11]. Additionally, there is clinical evidence of oxidative stress in the biological fluid of RP patients. In ocular samples of RP patients, elevated content of the oxidative stress markers CAR and 8-oxo-dG and decreased activity of the antioxidant enzyme extracellular superoxide dismutase (SOD3) are found [9,12,13].

Oxidative stress and inflammation are closely related pathophysiological processes. Chronic and sustained inflammation is observed during RP progression in animal models of RP and patients. The inflammatory process is accompanied by microglia activation, poly ADP ribose polymerase (PARP) activation, reactive gliosis, upregulation of cytokines such as the tumor necrosis factor-alpha (TNFα), etc. [14].

Nutraceuticals or functional foods are natural substances with health benefits through their antioxidant or anti-inflammatory properties, among others. They include polyunsaturated fatty acids (PUFAs), carotenoids (lutein, zeaxanthin), flavonoids (naringenin, quercetin, etc.), organic compounds (vitamins, polyphenols, etc.), minerals (Se, Zn, Cu, etc.), etc. Several studies support the protective role of nutraceuticals in ocular health [15]. They have been used as putative therapeutic strategies against different retinal diseases including RP, retinopathy of prematurity, or diabetic retinopathy [16,17,18,19]. In particular, nutritional supplementations such as *N*-acetylcysteine, resveratrol, or curcumin have been tested to delay retinal degeneration reducing oxidative stress and inflammatory mediators in preclinical models of RP, and to a lesser extent, in RP patients [2]. However, the efficacy of these nutritional supplementations on the progression of the RP seems to be controversial because of the different dosages, dosage regimen, mixtures of antioxidants, analyzed variables, number of participants, animal model of RP involved, etc. [20].

The aim of this study was to evaluate the efficacy of a mixture of nutraceuticals on the retinal function, the redox status, and the inflammatory process in rd10 mice, a model of RP.

## 2. Methods

### 2.1. Animals and Treatment

rd10 mice were used as a mouse model of autosomal recessive RP. Wild-type *C57Bl/6J* mice with the same genetic background as rd10 mice were used as a control group. Mice were kept under a 12 h light/dark cycle, humidity and temperature controlled. Mothers were fed with a standard chow diet (Table 1) and water ad libitum. All cages were placed on the lower shelf of an IVC rack with light illuminance of 115 ± 7 lux (95% CI: 98–131). Mice were housed in the Animal Facility of Research Center Principe Felipe (CIPF) or in the Unitat Central d’Investigació (UCIM) of Valencia University. At least eight animals for each group were used for each type of study (electroretinography, histological evaluation, redox status, and gene expression).

Based on several commercial formulations for humans, a mixture of nutraceuticals containing folic acid (vitamin B9), vitamin B6, vitamin A, zinc (Zn), copper (Cu), selenium (Se), lutein, and zeaxanthin was designed by Dr. José Miguel Soriano (Faculty of Pharmacy, Valencia University, Spain). This formulation, abbreviated NUT, was designed according to the Dietary Reference Intakes (DRI) for the Spanish Population for these compounds in humans [21]. In order to select the equivalent dosage for mice, we applied the formula for dose conversion between humans and mice (HED = NOAEL × Km ratio (for mice)) for each component [22]. Once a week, the formulation NUT was freshly prepared by dissolving the component powder in olive oil in order to obtain the desired final dose (in mg/kg): 0.052 folic acid, 0.523 vitamin B6, 0.244 vitamin A (retinol acetate), 2.787 Zn, 0.697 Cu, 0.012 Se, 2.787 lutein, and 6.62 zeaxanthin. rd10 pups received NUT every two days from postnatal day (PD) 9 until PD18. The mixture was dispensed using a pipette tip as follows: 3 µL of mixture per gram of body weight of the mouse (3 µL/g). Body weight was between 5 g and 9 g depending on the postnatal day and the litter size. This method allowed the administration of an exact dose. Some rd10 pups received the vehicle (olive oil, 3 µL/g) in order to control the effect of oil. However, we did not observe a significant effect of the vehicle (data not shown). Pups were euthanized by cervical dislocation at PD18. No apparent side effects were detected in animals treated with the mixture. For redox and gene expression studies retinas were isolated, placed immediately into the appropriate buffer, and stored at −80 °C.

### 2.2. ERG Recordings

Global retinal function was examined with scotopic full-field electroretinography (ERG) on 10 untreated rd10 mice, 15 rd10 mice treated with NUT, and 8 wild-type (*C57BL/6J*) mice at PD18. All experiments were performed under dim red light in a dark room. Mice were dark-adapted for at least 12 h before the experiments. Mice were anesthetized with the inhalational anesthetic isoflurane. Pupillary dilatation was achieved by topical application of a tropicamide 1%/eye drop (Alcon Cusí, Barcelona, Spain). To keep body temperature at 38 °C, mice were placed on a temperature-controlled heating table. Electrophysiological signals were recorded through electrodes placed inside the lower eyelids. To prevent the clouding of ocular media, the cornea was irrigated several times with saline solution. Two reference electrodes were inserted subcutaneously, one at the base of the tail (ground electrode) and the other at the level of the neck. The electrodes were connected to a two-channel amplifier. The responses to different light flashes (ranging from 0.0003 to 25 cd-s/m^2^) were averaged, amplified, and stored in a RetiScan-RetiPort electrophysiology unit (Roland Consult, Brandenburg an der Havel, Germany). The light stimulation device consisted of a Ganzfeld stimulator, which allowed a full-field retinal stimulation (RETIport scan/21, Roland Consult, Brandenburg an der Havel, Germany). Responses were collected simultaneously from both eyes. Before ERG was recorded, impedance and baseline tests were performed, the latter of which evaluated the noise level in the environment. The scotopic responses that primarily reflect rod function were evoked by flashes with low intensities (~0.01 cd-s/m^2^). Total retinal responses (rod plus cone function) were evoked by flashes with high intensities (≥3 cd-s/m^2^). For each light intensity, a series of ten ERG responses were averaged, and the interval between light flashes was adjusted to 10 s that allowed recovering responses. The RETI scan system amplifier collected the ERG data with a sampling rate of 2 kHz, which was then analyzed with the RETIport software (Roland Consult, Brandenburg an der Havel, Germany). The ERG response consisted of an initial negative component (a-wave) and ensuing positive peak (b-wave) evoked by light stimulation. The distance between the baseline and the first negative peak determined the amplitude of the a-wave. The amplitude of the b-wave was determined by measuring the distance between the peak of the a-wave and the next largest positive peak. The latency (implicit time) of a- and b-waves was determined by measuring the time from the start of the stimulus to the peak of each wave, respectively. The electrical activity of retinal cells was represented as the mean ± standard error of the mean (SEM) of b-wave amplitude for each light flash under scotopic conditions.

### 2.3. Retinal Histology

To obtain retinal sections, the eyes were rapidly removed and fixed in 4% filtered paraformaldehyde (#158127, Sigma-Aldrich, Madrid, Spain) for two hours at room temperature and cryoprotected in a sucrose gradient (15–20–30%). Eyes were frozen, embedded in frozen section compound (FSC22 Clear, Leica Biosystems, Richmond, VA, USA), nd 10 μm sections were cut in a cryostat (Leica CM1900, Leica Biosystems Nussloch GmbH, Nussloch, Germany). To evaluate cell death, the terminal deoxynucleotidyl transferase dUTP nick and labeling (TUNEL) assay (#G3250, Promega, Madison, WI, USA) was performed following the manufacturer’s instructions. For measurement of the number of nuclei at the outer nuclear layer (ONL), cryosections were stained with DAPI (#D9542, Sigma-Aldrich, Madrid, Spain).

For immunofluorescent staining procedures, cryosections were postfixed in 4% paraformaldehyde in 0.1 M phosphate buffer at pH 7.4 at room temperature for 15 min. For the recovery of epitopes, the cryosections were pretreated with citrate buffer pH 6.0. Cryosections were incubated with a blocking solution containing 1% bovine serum albumin, 5% normal goat serum, and 0.25% Triton X-100. (#A1388, Panreac Applichem, Darmstadt, Germany) for one hour. Then, they were incubated with the primary antibody against ionized calcium-binding adaptor protein-1 (Iba1) (1:300, #019-19741, Wako Pure Chemical Industries Ltd., Osaka, Japan), glial fibrillary acidic protein (GFAP) (1:400, #G3893, Sigma-Aldrich, Madrid, Spain), or poly-ADP ribose polymers (PAR) (1:100, #ALX-804-220, Enzo Life Science, Madrid, Spain) overnight at 4 °C. Cryosections were incubated with the secondary antibodies conjugated with fluorescence Alexa Fluor 488 or 647 (1:400, #A-11001, #A-21235, Invitrogen, Life Technologies, Madrid, Spain) at room temperature for one hour. A counterstaining was performed with DAPI, and cryosections were mounted on Fluoromount-G (#0100-01, Southern Biotechnology, Birmingham, AL, USA and visualized under a TCS SP5 confocal microscope (Leica Microsystems CMS GmbH, Mannheim, Germany) at 22 °C. Eight retinas were analyzed for each group. Slides without primary antibodies served as a negative control.

### 2.4. Microscopy and Quantification

We used a TCS SP5 confocal microscope from the Microscope Unit of IIS-La Fe (Valencia, Spain) with a spatial resolution of 1024 × 1024 to examine the retinal cryosections. Fixing the pinhole at 1 airy unit and doing z-stacks of 10 pictures (1.0 μm steps), z-stacks were performed under 63× magnification, with an acquisition rate of 16 frames per second. A zoom factor of 4.0 or 6.0 was employed in some retinal cryosections. Leica LAS AF was used as microscope imaging software (Leica Microsystems CMS GmbH, Mannheim, Germany). Negative controls (without primary antibodies) were used to detect auto fluorescence or noise. The acquisition parameters for each fluorophore were adjusted (e.g., gain, smart offset, and excitation energy) to obtain a proper image. In the case of double immunostaining (Iba1 and PAR staining) plus DAPI counterstain sequential acquisition was used to avoid crosstalk between fluorophores. Direct counting of photoreceptor nuclei in the ONL, the number of TUNEL-, PAR- and Iba1-positive cells (manual cell counting), and the integrated density of GFAP was carried out using the ImageJ open source software. Adobe Photoshop 10 software (Adobe Systems Inc., San Jose, CA, USA) was used to process the final images. We measured the ONL thickness as the number of nuclei of photoreceptors. In the rd10 model, the ONL thickness and the degenerative process are different along the retina, for this reason, we performed several measurements of nuclei in ONL across the entire retina (from the nasal to the temporal retina) for each mouse. At least eight entire retinas were analyzed per experimental group.

The number of TUNEL- and PAR-positive cells was represented as the ratio between the positive cells and the inner nuclear layer (INL) area or the total retinal area, respectively. Microglial activation (migration index) was measured as previously described [11]. Corrected fluorescence of GFAP was quantified as previously described [23]. TUNEL-, and PAR-positive cells, microglial migration index, and the corrected fluorescence of GFAP were quantified from six nonadjacent sections of at least eight retinas for each experimental group.

### 2.5. Determination of Antioxidant and Oxidant Markers

Ocular redox status was evaluated by measuring total antioxidant capacity (TAC), activities of cytosolic superoxide dismutase (SOD1), mitochondrial SOD (SOD2), catalase (CAT), and glutathione peroxidase (GPx), protein carbonyl content (CAR), and formation of thiobarbituric acid reactive substances (TBARS, indicator of lipid peroxidation) in retinal tissue from at least eight retinas for each experimental group. As previously described [11], retinas were homogenized in 5 mM phosphate buffer pH 7, 0.9% NaCl, 0.1% glucose, centrifuged at 10,000× *g* for 15 min at 4 °C.

Specifically, TAC was measured by detecting the oxidation of 2,2′-azino-di-[3-ethylbenzthiazoline sulphonate] (ABTS) by metmyoglobin (absorbance at 405 nm) (#709001, Cayman Chemical, Ann Arbor, MI, USA). TAC levels were expressed as nmol of antioxidants/mg protein. Determination of SOD1 and SOD2 activities were based on the dismutation of superoxide oxygen and hydrogen peroxide with a commercial kit. A tetrazolium salt reacted with superoxide radicals generated by xanthine oxidase and hypoxanthine and the formazan dye was colorimetrically measured (absorbance al 450 nm) (#706002, Cayman Chemical, Ann Arbor, MI, USA). To separate SOD1 from SOD2 retinal homogenates were centrifuged at 10,000× *g* for 15 min at 4 °C. The resulting 10,000× *g* supernatant contained SOD1 and the pellet contained SOD2. SOD activities were expressed as U/mg protein. Determination of CAT activity was based on the reaction of catalase with methanol in the presence of H_2_O_2_. The formaldehyde produced was measured colorimetrically with 4-amino-3-hydrazino-5-mercapto-1,2,4-triazole (absorbance at 540 nm) (#707002, Cayman Chemical, Ann Arbor, MI, USA). CAT activity was expressed as nmol of formaldehyde/min.mg protein. Determination of GPx activity was measured indirectly by a coupled reaction with glutathione reductase (GR). Oxidized glutathione produced by GPx was recycled to its reduced state by GR and NADPH. The oxidation of NADPH to NADP+ was colorimetrically measured (absorbance at 340 nm) (#703102, Cayman Chemical, Ann Arbor, MI, USA). GPx activity was expressed as nmol/min.mg protein.

Lipid peroxidation was evaluated by measuring the formation of TBARS (including malondialdehyde, MDA), which were formed as a byproduct of lipid peroxidation (#10009055, Cayman Chemical, Ann Arbor, MI, USA). TBARS levels were expressed as nmol of MDA/mg protein. CAR was measured using fluorescein-5-thiosemicarbazide (FTC), a fluorescent probe that covalently reacted with oxidized residues on proteins. FTC generated a stable fluorometric signal that was monitored (Ex/Em 485/535 nm) (#ab235631, Abcam, Cambridge, UK). CAR content was expressed as nmol/mg protein.

Protein concentration was measured by the bicinchoninic acid (BCA) protein assay (#23225, BCA Kit; Pierce Scientific, CA, USA). Protein content was expressed as mg/mL.

### 2.6. Gene Expression Analysis

Total RNA was isolated from frozen retinas at PD18 (eight retinas for each experimental group) using the NZY Total RNA Isolation Kit (#MB13402, Nzytech, Lisboa, Portugal), following the manufacturer’s protocol. RNA concentration was determined by spectrophotometry on the NanoDrop 2000 (Thermo Fisher Scientific, Wilmington, DE, USA). Then, cDNA was synthesized starting from 0.5 μg of RNA by reverse transcription using the PrimeScript™ RT Reagent Kit (Perfect Real Time) (#RR037A, Takara-Bio, Otsu, Japan), following the manufacturer’s instructions. The cycling conditions consisted of reverse transcription at 37 °C for 15 min and inactivation of reverse transcriptase at 85 °C for five seconds.

The relative expression of TNFα, TNFR1, IL-6, IL-1β, IL-18, and GFAP was measured in retinas by real-time PCR using thermal cycler (LightCycler^®^ 480 System; Roche, Basel, Switzerland), TaqMan gene expression assay, specific TaqMan probes: Mm00443260_g1 (TNF-α), Mm00441883_g1 (TNFR1), Mm01253033_m1 (GFAP), Mm00434228_m1 (IL-1β), Mm00434226_m1 (IL-18), Mm00446190_m1 (IL-6), and Premix Ex Taq master mix for probe-based, real-time PCR (#RR390A, Takara-Bio, Otsu, Japan). β2-microglobulin (β2m) gene (Mm00437762_m1) was used as the housekeeping gene. Real-time PCR was performed with one cycle of denaturation of 30 s at 95 °C, continued by 40 cycles of five seconds denaturation at 95 °C, 30 s annealing at 60 °C, and one cycle of extension at 50 °C for 30 s. Relative gene expression was normalized with the housekeeping gene. Then, normalized values of control mice were normalized to one to determine the changes in the gene expression of untreated rd10 mice and NUT-treated rd10 mice.

### 2.7. Statistical Analysis

Statistical analyses were performed using Graph Pad Software 9.0 (Prism; Graph Pad Software, Inc., San Diego, CA, USA). Normal distribution of data was analyzed by Shapiro–Wilk and Kolmogorov–Smirnov tests. Comparisons between control, rd10, and rd10 + NUT groups were performed using one-way ANOVA and post hoc Tukey’s multiple comparisons test or Kruskal–Wallis and post hoc Dunn’s multiple comparisons test depending on data distribution (parametric or nonparametric analysis). In some cases, comparisons between rd10 and rd10 + NUT groups were performed using Mann–Whitney test. For each analysis, 8 to 15 animals/group were used. A *p* value < 0.05 was considered statistically significant. The data were plotted using Graph Pad Software 9.0. The data were presented as mean ± SEM.

## 3. Results

### 3.1. Oral Administration of Antioxidant Nutraceuticals Ameliorated Retinal Degeneration in rd10 Mice at PD18

**Figure 1:**
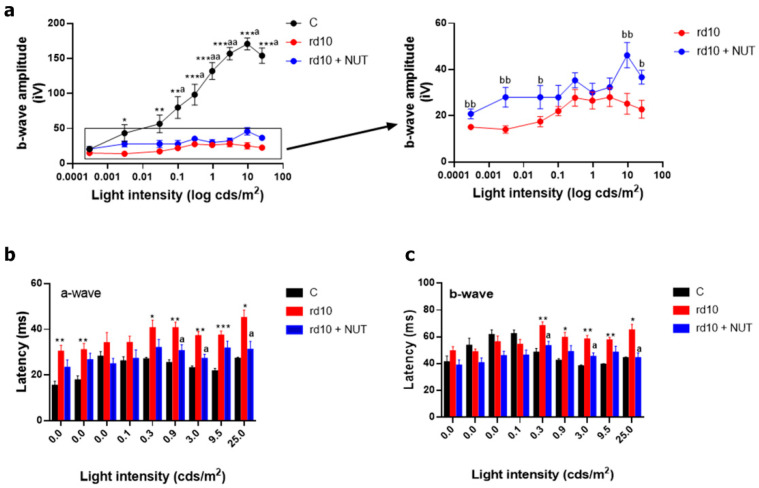
Effect of oral administration of nutraceuticals on retinal degeneration in rd10 retinas at PD18: (**a**) amplitudes of ERG b-wave; (**b**) a-wave implicit time or latency; (**c**) b-wave implicit time or latency recorded from dark-adapted PD18 control mice (C), untreated rd10 mice and NUT-treated rd10 mice at different intensities of light stimuli. rd10 mice were treated with NUT from PD9 to PD18. Kruskal–Wallis test and post hoc Dunn´s multiple comparisons test to compare three groups or Mann–Whitney test to compare rd10 vs. rd10 + NUT. * *p* < 0.05; ** *p* < 0.01; *** *p* < 0.0001 for differences between control and rd10 mice; ^a^ *p* < 0.05; ^aa^ *p* < 0.01 for differences between control and rd10 + NUT mice; and ^b^ *p* < 0.05; ^bb^ *p* < 0.01 for differences between rd10 and rd10 + NUT mice. Data were presented as mean ± standard error of the mean (SEM). At least eight mice are analyzed for each group.

We previously reported the first peak of photoreceptor degeneration (rods) and an altered antioxidant response in rd10 retinas at PD18 [10,11]. Moreover, other authors showed altered retinal function at this age [24]. In the current study, we determined whether NUT supplementation had neuroprotective effects on retinal degeneration evaluating the function and morphology of rd10 retinas without or with NUT. Firstly, we assessed the visual function of untreated rd10 mice by registering the global electrical response of the retina to different light stimuli. As expected, global ERG responses were not well preserved in the retina of these animals. Scotopic ERG recordings showed a significant decrease in b-wave amplitudes at multiple flash intensities, compared with control mice (*p* < 0.05, Kruskal–Wallis and post hoc Dunn´s multiple comparisons) (Figure 1a). The a-wave was hardly detectable.

The a-wave implicit time or latency of control mice (about 15–28 ms) was faster than in rd10 mice (about 30–45 ms) for most intensities used (*p* < 0.05, Kruskal–Wallis test, and post hoc Dunn´s multiple comparisons test) (Figure 1b). The b-wave implicit time or latency of control mice (about 40∼48 ms) was also faster than in rd10 mice (about 55–70 ms) for the highest intensities used (0.3–25 cds/m^2^) (*p* < 0.05, Kruskal–Wallis test and post hoc Dunn´s multiple comparisons test) (Figure 1c). Therefore, we evaluated whether NUT ameliorated visual dysfunction. Based on ERG responses, amplitudes of scotopic b-wave were slightly higher in NUT-treated rd10 mice than in untreated rd10 mice for several light intensities (*p* < 0.05, Mann–Whitney test). a- and b-wave implicit times also improved for the highest intensities (*p* < 0.05, Kruskal–Wallis test, and post hoc Dunn´s multiple comparisons test). Thus, NUT partially recovered retinal function in rd10 mice.

**Figure 2:**
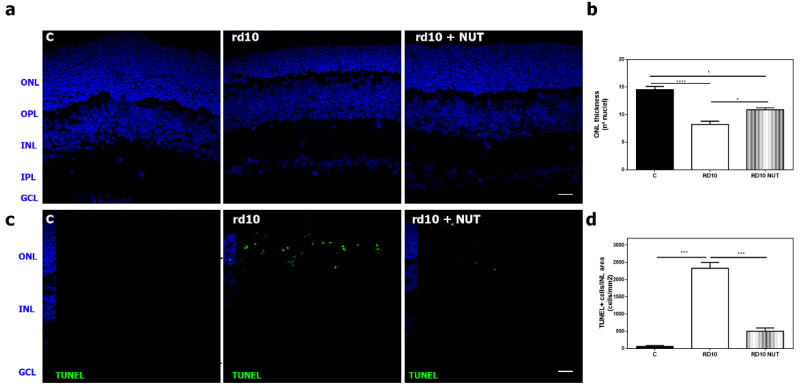
Effect of oral administration of nutraceuticals on retinal degeneration in rd10 retinas at PD18: (**a**,**b**) representative photomicrographs of retinal sections showing DAPI staining and (**b**) quantification of number of rows of nuclei in the ONL in control mice (C), untreated rd10 mice and NUT-treated rd10 mice; (**c**,**d**) representative photomicrographs of retinal sections showing TUNEL-stained and DAPI-counterstained sections and (**d**) quantification of the number of TUNEL-positive nuclei cells in control mice (C), untreated rd10 mice and NUT-treated rd10 mice. rd10 mice were treated with NUT from PD9 to PD18. Scale bar: 20 µm. ONL: outer nuclear layer; OPL: outer plexiform layer; INL: inner nuclear layer; IPL: inner plexiform layer; GCL: ganglion cell layer. Kruskal–Wallis test and post hoc Dunn’s multiple comparisons test, * *p* < 0.05; *** *p* < 0.001; **** *p* < 0.0001. Data were presented as mean ± standard error of the mean (SEM). Eight–ten mice were used for each group.

Secondly, we assessed a histological evaluation by measuring the number of the remaining photoreceptors (number of rows of nuclei at ONL), and the number of TUNEL positive cells (DNA fragmentation of dying cells mainly apoptotic cells) (Figure 2). At PD18, NUT significantly increased the number of rows of nuclei at ONL in rd10 mice, compared with untreated rd10 mice (*p* = 0.015, Kruskal–Wallis test and post hoc Dunn’s multiple comparisons test) (Figure 2a,b). ONL thickness was better preserved after NUT supplementation. TUNEL assay that detected DNA fragmentation (a late event of cell death) showed that the number of TUNEL positive cells at the ONL was significantly lower in NUT-treated rd10 mice than in untreated rd10 mice (*p* < 0. 0001, Kruskal–Wallis test and post hoc Dunn´s multiple comparisons test) (Figure 2c,d).

### 3.2. Oral Administration of Antioxidant Nutraceuticals Partially Restored Retinal Redox Status in rd10 Mice at P18

**Figure 3:**
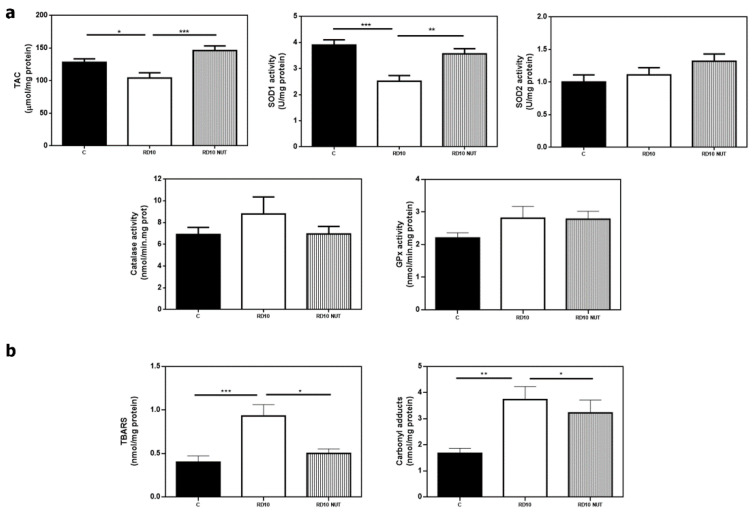
Effect of oral administration of nutraceuticals on retinal redox status in rd10 mice at PD18. (**a**) Values of the antioxidant markers TAC, and activities of superoxide dismutase (SOD) 1 or cytosolic (SOD1), 2 or mitochondrial (SOD2), catalase (CAT) and glutathione peroxidase (GPx), and (**b**) values of the oxidant markers thiobarbituric acid reactive substances (TBARS, indicator of lipid peroxidation) and protein carbonyl groups (CAR, indicator of protein oxidation) from control mice (C), untreated rd10 mice and NUT-treated rd10 mice. rd10 mice were treated with NUT from PD9 to PD18. Data were presented as mean ± S.E.M. from at least eight retinas for each experimental group. Statistical differences between groups (*p* < 0.05) were shown * *p* < 0.05; ** *p* < 0.01; *** *p* < 0.001 using one-way ANOVA or Kruskal–Wallis test and post hoc Tukey’s or Dunn´s multiple comparisons tests.

We previously described that antioxidant response was reduced in rd10 retinas at PD18 [10,11]. Moreover, we hypothesized that rd10 retinas were exposed to a hyperoxic environment that would contribute to free radical generation and oxidative damage in lipids, proteins, etc. [12]. In the current study, we confirmed an altered retinal redox status in rd10 mice with a significant decrease of the antioxidant markers TAC, and SOD1 activity (*p* < 0.05 and *p* = 0.0002, respectively, Kruskal–Wallis test and post hoc Dunn’s multiple comparisons test) (Figure 3a), and a significant increase of the oxidant markers TBARS and CAR (*p* = 0.0005, *p* = 0.0045, respectively, Kruskal–Wallis test, and post hoc Dunn’s multiple comparisons test), compared with control mice (Figure 3b). The activities of the other antioxidant enzymes SOD2, CAT, and GPx did not significantly change (*p* > 0.05, one-way ANOVA and post hoc Tukey’s multiple comparisons tests, for SOD2 and CAT, and Kruskal–Wallis test and post hoc Dunn’s multiple comparisons test for GPx) (Figure 3a).

Oral supplementation with NUT partially restored retinal redox status. NUT-treated rd10 mice showed higher values of TAC and SOD1 activity than untreated rd10 mice (*p* = 0.0009, *p* = 0.0091, respectively; Kruskal–Wallis test and post hoc Dunn’s multiple comparisons test) (Figure 3a). NUT treatment also reduced TBARS formation (a marker of lipid peroxidation) (*p* = 0.037, Kruskal–Wallis test and post hoc Dunn’s multiple comparisons test) but had no statistical effect on CAR content (a marker of protein oxidation) (Figure 3b).

### 3.3. Oral Administration of Antioxidant Nutraceuticals Reduced Retinal Inflammation in rd10 Mice at PD18

**Figure 4:**
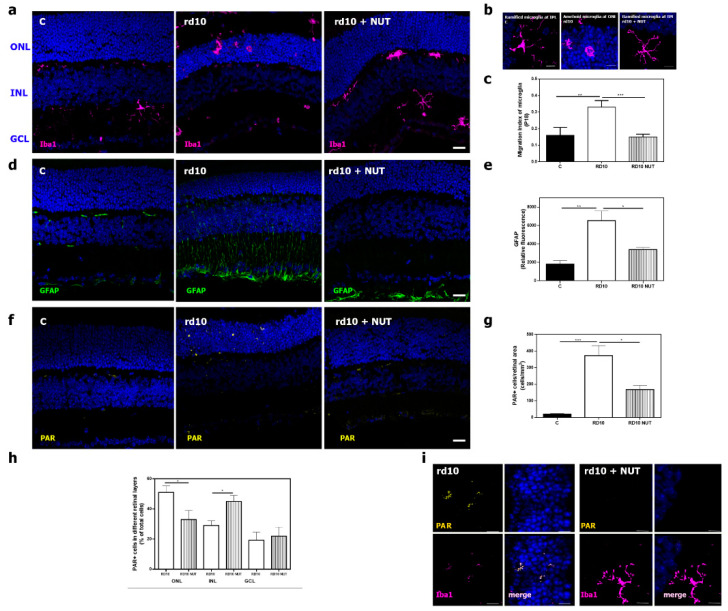
Effect of oral administration of nutraceuticals on retinal inflammation in rd10 mice at PD18: (**a**) representative photomicrographs of retinal sections showing Iba1-labeling (microglial cells) in DAPI-counterstained section from control mice (C), untreated rd10 mice, and NUT-treated rd10 mice. rd10 were treated with NUT from PD9 to PD18, scale: 20 µm; (**b**) optical zoom showing amoeboid and ramified shape of microglia cells in retinas of untreated rd10 mice and NUT-treated rd10 mice, scale: 10 µm; (**c**) index of microglial migration in these retinas; (**d**,**e**) representative photomicrographs of retinal sections showing GFAP-labeling (Müller cells) in DAPI-counterstained sections and corrected fluorescence from control (C), untreated rd10 and NUT-treated rd10 mice, scale: 20 µm; (**f**,**g**) representative photomicrographs of retinal sections showing poly ADP-ribose polymers (PAR) accumulation, quantification of PAR positive cells from control (C), untreated rd10 and NUT-treated rd10 mice scale: 20 µm; (**h**) quantification of the distribution of PAR positive cells throughout the retina of untreated rd10 and NUT-treated rd10 mice, (**i**) optical zoom showing double-immunostaining of Iba1 and PAR in retinas of untreated rd10 mice and NUT-treated rd10 mice, scale: 5 µm. ONL: outer nuclear layer; INL: inner nuclear layer; GCL: ganglion cell layer. Data were presented as mean ± SEM. from at least eight retinas for each experimental group. Statistical differences between groups (*p* < 0.05) were shown * *p* < 0.05; ** *p* < 0.01; *** *p* < 0.001 using one-way ANOVA or Kruskal–Wallis test and post hoc Tukey’s or Dunn’s multiple comparisons tests.

As mentioned above, oxidative stress and inflammation are tightly interrelated cellular processes. We previously observed that rd10 retinas showed several markers of inflammation including microglia activation, which led microglia cells to migrate from the inner to the outer retina changing their morphology, reactive gliosis (upregulation of GFAP, an intermediate filament protein), or upregulation of cytokines such as TNFα. In addition, inflammation was related to poly (ADP) ribose polymerase over activation and subsequent polyADP ribose (PAR) polymers accumulation [11,25]. Oral administration of NUT ameliorated retinal inflammation in rd10 mice, compared with untreated rd10 mice. We labeled microglia cells with an antibody against Iba1, and we calculated the migration index (MI) of these cells from the inner to the outer retina, as previously described [10]. As shown in Figure 4a–c, retinas from control mice presented Iba-1 positive cells (microglia) with a ramified shape, which were localized close to the inner retina (quiescent cells). Retinas from untreated rd10 mice show Iba-1 positive cells with amoeboid shape and migration to the outer retina where photoreceptors were localized. Retinas from NUT-treated rd10 mice presented Iba-1 positive cells with a more ramified shape than untreated rd10 retinas, and they were located closer to the inner retina. A significant reduction of MI was observed in NUT-treated rd10 mice, compared with untreated rd10 mice (*p* = 0.0006, one-way ANOVA and Tukey’s multiple comparisons tests) (Figure 4c). We confirmed reactive gliosis with upregulation of GFAP in rd10 mice, compared with control mice (*p* = 0.0017; Kruskal–Wallis test and post hoc Dunn’s multiple comparisons test). NUT significantly reduced GFAP upregulation, a hallmark for reactive gliosis, in NUT-treated rd10 mice (*p* = 0.041; Kruskal–Wallis test and post hoc Dunn’s multiple comparisons test) (Figure 4d,e). Gene expression studies also confirmed the findings in GFAP content (Table 2).

The number of PAR-positive cells was increased in untreated rd10 mice, compared with control mice. NUT significantly reduced the number of cells that accumulated PAR in rd10 mice (*p* = 0.0003; Kruskal–Wallis test and post hoc Dunn’s multiple comparisons test) (Figure 4f,g). In particular, we also observed changes in the distribution of PAR-positive cells throughout the retina of NUT-treated rd10 mice, compared with the retina of untreated rd10 mice (Figure 4h). Most PAR-positive cells were localized at ONL in untreated rd10 mice, while PAR-positive cells were mainly localized at INL in NUT-treated rd10 mice (*p* < 0.05; Mann–Whitney test). Finally, we corroborated previous data showing that microglia cells presented accumulation of PAR (an indicator of PARP over activation) polymers with double staining of Iba1 and PAR in rd10 mice (Figure 4i).

Upregulation of the cytokines TNFα, IL-6, and IL-1β and downregulation of IL-18 was previously described in rd10 retinas at PD18 or PD23 [10,11,25]. In this study, we corroborated upregulation of TNFα, IL-6, and IL-1β in untreated rd10 mice with respect to control mice (*p* < 0.05; Kruskal–Wallis test and post hoc Dunn´s multiple comparisons test) at PD18 (Table 2). In addition, we observed lower gene expression of IL-18 in untreated rd10 mice than in control mice. TNFR1 expression was also upregulated in rd10 mice at this age. We showed that NUT supplementation was capable to modulate the inflammatory profile reducing IL-6, IL-1β, and TNFR1 (*p* = 0.005, *p* = 0.0098 and *p* = 0.0004, respectively), and to a lesser extent, TNFα (*p* = 0.12) expression in rd10 mice (Table 2).

## 4. Discussion

Currently, there are no effective treatments available to address the progression of inherited retinal disorders such as RP, except for RPE65 gene therapy (voretigene neparvovecrzyl, Luxturna; Spark Therapeutics). RP exhibits a high clinical and genetic heterogeneity, which makes it difficult to find an appropriate treatment. There are several therapeutic approaches to treat it depending on its progression. These approaches include retinal prosthesis, gene therapy, cell therapy, or pharmacological therapy. The latter focuses on reducing the loss of photoreceptor cells and subsequent loss of vision, independently of the genetic defect. In spite of being a genetic disease, many studies confirm the role of oxidative stress and inflammation in photoreceptor cell death [8,26,27]. Therefore, the use of antioxidant or anti-inflammatory compounds is a putative therapeutic strategy. For instance, previous studies suggest that strategies targeting oxidative stress through antioxidants are effective in slowing down cone degeneration and to a lesser extent rod degeneration [26,27,28].

In the current study, we assessed a therapeutic approach based on nutraceuticals with antioxidant and anti-inflammatory properties that could slow down retinal degeneration in rd10 mice, a model of autosomal recessive RP. Nutraceutical formulations still show limited benefits, their efficacy should be improved, and the health benefits remain controversial. The formulation NUT contained folic acid, vitamin B6, vitamin A, Zn, Cu, Se, lutein, and zeaxanthin. Some components of the formulation NUT previously showed beneficial effects when they were separately administered. In the market, two commercial formulations exist with relatively high doses of vitamin C, E, Cu, and Zn plus beta-carotene (AREDS) or lutein/zeaxanthin (AREDS2) [16,29,30,31], which show benefits for patients with intermediate or late age-related macular degeneration (AMD) [32,33]. However, the effect of the proposed formulation (NUT) had not been already tested in animal models or patients.

Vitamin A is a group of unsaturated organic nutritional compounds including provitamin A, retinal, retinoic acid, carotenoids, and retinol. Vitamin A is involved in many ocular functions including rod function and dark adaptation [34]. In particular, vitamin A participates in the visual cycle, which converts vitamin A (all-trans-retinol) into 11-cis-retinal, the light-sensitive visual chromophore involved in the phototransduction process [35]. Evidence suggests that vitamin A deficiency causes night blindness and structural degeneration of the retina [36]. It has been reported a beneficial effect of vitamin A on the course of RP in patients based on retinal responses to light [37]. However, it is important to highlight that vitamin A supplementation may cause adverse effects in humans. Accumulation of toxic by-products of vitamin A in the retina is a risk factor for young RP patients or patients with ABCA4 mutations [38].

Vitamins B6 and B9 (folic acid) seem to have a beneficial effect on ocular health. They have antioxidant properties via multiple mechanisms [39]. Vitamin B6 acts as a coenzyme in the glutathione system, and it also reacts with peroxy radicals as scavenger-inhibiting lipid peroxidation [40]. Folic acid reduces ROS formation, increases TAC, and reduces homocysteine levels in plasma [41]. Their combination seems to have a protective role in AMD reducing the risk of its appearance or progression [42,43]. Recently, vitamin B6 prevented retinal neovascularization through the inhibition of HIF activation and subsequent downregulation of VEGF in an AMD model [44]. A mixture of nutraceuticals containing vitamin B2, B6, B1, B12, etc. efficiently counteracted ganglion cell degeneration and inflammation (cytokine release, GFAP upregulation, and microglia activation) in two different mouse models of glaucoma [45,46]. To our knowledge, folic acid supplementation has not been already tested for RP. However, administration of folic acid downregulated inflammatory molecules (IL-1β and NLRP3) and oxidative markers in a mouse model of diabetic retinopathy [47].

The minerals Zn, Se, and Cu are essential trace elements with key roles in cellular antioxidant defense processes [48,49,50]. It is difficult to find studies with mineral supplements such as Zn, Cu, or Se alone in ocular diseases. Due to their properties, they are part of a complex formulation that includes other compounds (e.g., vitamins) [51]. After iron, the most abundant metal in the human body is Zn. It binds to metallothioneins (MTs) participating in the defense against oxidative damage and inflammation [52]. Cell destruction induced by oxidative damage is mitigated by MTs, which are responsible for capturing and neutralizing free radicals owing to cysteine sulfide ligands [53]. Apart from its antioxidant role, Zn is essential in the retina because it interacts with taurine and vitamin A modifying photoreceptor membranes, regulating synaptic transmission and light-rhodopsin reaction [54]. Zn depletion promotes reduced dark adaptation and electrical response of the retina. Zn deficiency was found in AMD eyes and their supplementation may have beneficial effects in AMD patients [55,56].

Se is an essential constituent of selenoproteins such as GPx or thioredoxin reductases, which have antioxidant and enzymatic capacities [57]. Se may regulate the inflammatory response by reducing TNFα release, cyclooxygenase 2, and nuclear factor κB (NF-κB) activation [58].

Cu is involved in eliminating superoxide radicals by the SOD enzyme. Cu is built-in in the active center of several enzymes including cytochrome c oxidase, ceruloplasmin, SOD1, or extracellular SOD3 [59]. Normal Cu metabolism is essential to ocular tissue, and its alteration is related to glaucoma or AMD [54,60]. Cu is necessary for the synthesis of melanin, a storage protein for Fe, Zn, and Cu in retinal pigment epithelium (RPE) and melanocytes. Zn and Cu are components of the AREDS and AREDS2 formula that recommended for patients with intermediate or late AMD. The literature suggested a slight effect of zinc on reducing AMD progression, but it did not find any significant effect of copper or selenium [61].

Lutein and zeaxanthin are dietary carotenoids that form the yellow macular pigment of the human eye. Evidence suggests that macular pigment protects retinal cells from oxidative damage by removal of ROS by de-excitation, reduction of other radicals, attenuation of blue light [62], and cell death in different retinopathies [63]. They ameliorated photoreceptor function probably via decreasing endoplasmic reticulum stress in a mouse model of RP and in a mouse model of light-induced retinopathy [64,65]. In addition, it is suggested that they may improve normal ocular function by reducing glare disability and increasing contrast sensitivity. In animal models of retinal degeneration, lutein reduced oxidative stress [66]. Lutein and zeaxanthin supplementations were associated with decreased AMD risk and less visual impairment [67,68]. In RP patients, lutein supplementation seemed to improve the visual field [68,69,70].

Apart from their antioxidant role, vitamin A, B6, folic acid, Cu, Zn, and Se present often synergistic roles during the immune response. They act on the differentiation, proliferation, functioning, and movement of natural killer cells, macrophages, and T cells. They help to modulate cytokine release including TNFα, IL-6, IL-2 [71]. In particular, lutein and zeaxanthin reduced cytokines (IL-1β, IL-6) and NF-κB levels probably activating the Nrf2 pathway in a mouse model of traumatic brain injury [72]. Vitamin A also has a role in enhancing immune function. Vitamin A in the form of retinoic acid regulates the function, differentiation, and maturation of innate immune cells. For instance, all-trans-retinol inhibited the release of inflammatory molecules from macrophages, inducing their polarization from M1 to M2 macrophages in the bone marrow to transform into M2 macrophages [73]. Retinoic acid also acts on adaptive immune cells by stimulation and proliferation of B cells or modulation of the differentiation of T cells [74].

Folic acid (vitamin B9) may reduce inflammation (IL-1β and NLRP3) in a mouse model of diabetic retinopathy [47]. Zn participates in the development of the immune system both innate and adaptive [75]. For instance, Zn supplementation inhibited complement activation in AMD [76].

Several studies suggest that the coadministration of two or more dietary supplements should be more effective than each individual one for several diseases [77,78,79,80]. We suggest a synergistic effect of NUT that could offer advantages including higher bioavailability, protection of their radical scavenging role, or their biological effects [81].

In previous studies, we observed that the degeneration of rd10 retinas was quite evident and it was accompanied by oxidative damage and inflammation including microglia activation, reactive gliosis, or upregulation of cytokines at PD18 or PD23 [11,25]. In the current study, ERG data showed that the global retinal response of rd10 mice was reduced with respect to control mice, as other authors previously reported [82]. We observed that oral administration of the formulation NUT ameliorated degeneration, oxidative damage, and inflammation at PD18, when we detected the first peak of photoreceptor degeneration [11]. We showed a functional and morphological recovery in retinas of NUT-treated rd10 mice. The retinal response to different light intensities was a bit higher after NUT treatment (b-wave amplitude results) than in untreated retinas. The implicit time of a- and b-wave also was improved by NUT treatment. Our results support the previous data published by other groups on the neuroprotective role of antioxidant molecules such as curcumin, naringenin, quercetin, progesterone, vitamin A, lutein, zeaxanthin, or lipoic acid in photoreceptor degeneration in animal models or RP patients [18,83,84,85].

As mentioned above, the formulation NUT contains antioxidant compounds that ameliorated the redox status found in the retinas of rd10 mice. The cellular mechanism by which antioxidants would protect the retina from degeneration would be through the inhibition of abnormal production of ROS, the neutralization of free radicals, and the augmentation of the antioxidant defense system [86]. We analyzed the main antioxidant enzymes (SOD, CAT, and GPX) responsible for the antioxidant defense, and the global antioxidant capacity (TAC) in rd10 mice with or without NUT supplementation. At PD18, the antioxidant defense seemed to be reduced (SOD1 and TAC) in rd10 mice without supplementation corroborating previous findings [11]. As previously mentioned, oxidative stress is the result of an imbalance in the production of ROS and the activity of the antioxidant defense machinery. Under pathological situations, ROS accumulate and compromise the cell function because they induce alteration of macromolecules such as fragmentation of DNA, lipid peroxidation, protein carbonylation, etc. In our case, downregulation of the antioxidant machinery was accompanied by an increase in lipid peroxidation (TBARS) and protein carbonylation (CAR). The antioxidant machinery includes endogenous antioxidant defense enzymes includes SOD, CAT, GPx, and glutathione reductase (GR), endogenous nonenzymatic factors such as vitamin C, vitamin E, or glutathione (GSH) [87], and exogenous antioxidants of natural origin such as lutein, polyphenols, flavonoids, etc. Exogenous antioxidants may act directly as scavengers of ROS, inducers of expression of antioxidant enzymes, or inhibitors of free radical chain reactions [88]. Nutraceuticals are nonenzymatic antioxidants that could alleviate the downregulation of the antioxidant machinery. In our case, oral supplementation with NUT ameliorated the imbalance by restoring TAC levels and SOD1 activity and reducing TBARS. However, NUT cannot reduce protein carbonylation (CAR). For instance, saffron supplementation also reduced MDA levels and increased GPx and SOD activities in the retinas of diabetic rats [89]. Resveratrol, a polyphenol compound, was capable to act as a free radical scavenger or modulator of the SOD activity reducing ROS formation and MDA levels in different retinopathies [90,91,92]. Lutein reduced the production of 4-HNE, a major end product of peroxidation of membrane PUFAs and protein carbonylation in retinal pigment epithelium of retina from mice lacking αvβ5 integrin [93].

Oxidative stress and inflammation are highly interconnected cellular processes. We showed that NUT supplementation reduced microglial activation (migration and morphology), IL-6, IL-1β, and to a lesser degree TNFα upregulation. Microglial activation is a hallmark of neuroinflammation. Under pathological conditions, ramified microglia (resting state) change their morphology and acquire an activation state [94]. They enlarge their cell body and shorten their ramifications, leading to amoeboid morphology. The activated microglia would move toward sites of injury, in this case, the photoreceptor layer, where they would release inflammatory mediators such as IL-1β, TNFα, etc. that would lead to increase oxidative damage and inflammation. Persistent microglia activation is harmful to nervous tissue including the retina; therefore, it could contribute to photoreceptor cell death including healthy cones by secreting neurotoxic factors (proinflammatory cytokines and chemokines). There are two phenotypes of activated microglia: M1 and M2. M1 microglia cells secrete proinflammatory molecules such as TNFα, IL-1β, or nitric oxide. M2 microglia cells secrete anti-inflammatory molecules contributing to repair the damage [95,96]. The shift to the different subtypes of M2 (2a–c) is driven by IL-3, IL-4, activation of Toll-like receptors (TLRs) agonist, IL-10, TGFβ1, or glucocorticoids [97]. It seems that microglia activation has a dual role, both neurotoxic and neuroprotective, depending on their state. Immunomodulation strategies aiming to reduce M1 microglia activation or to induce the shift from M1 to M2 phenotype may be useful to increase photoreceptor survival. These strategies can be based on drugs (pharmacological approaches) or nutrients (nutritional approaches) [98]. Some studies suggest that nutritional approaches would ameliorate microglial activation in different retinopathies. For instance, DHA supplementation enhanced photoreceptor survival and reduces microglial activation in a mouse model of inherited retinal degeneration. In addition, DHA decreased proinflammatory genes and migration in BV2 microglia [99]. Lutein supplementation improved retinal function and reduced microglial activation in a murine model of diabetic retinopathy [100]. Curcumin was capable to improve retinal degeneration and reduce microglial activation in *rd1* mice, a model of RP [83]. In addition, it was capable to switch the M1 phenotype to the M2 phenotype decreasing proinflammatory molecules in LPS-treated BV2 microglial cells [101]. Saffron extract ameliorated retinal ganglion cells and reduced microglial activation in a model of glaucoma [102]. Therefore, nutraceutical strategies aimed to immunomodulate, e.g., polarization M1/M2 microglia, would be useful to treat many retinopathies including RP, diabetic retinopathy, AMD, or glaucoma.

As described above, the retina is highly susceptible to oxidative stress. Accumulation of ROS may cause DNA fragmentation, resulting in PARP activation [25]. NUT supplementation decreased PARP activation, reducing the accumulation of PAR polymers at ONL. PARP-1, the most abundant isoform of the PARP enzyme family, is implicated in the modulation of inflammatory pathways, transcription, chromatin remodeling, etc. by PARylation. PARylation is a post-translational modification by covalent attachment of PAR polymers to histones, DNA-associated proteins, etc. In addition, PARP-1 stimulates the production of PAR polymers and the release of apoptosis-inducing factor (AIF) from the mitochondria, leading to a type of cell death called PARthanatos [103]. Accumulating evidence reveals that excessive activation of PARP-1 contributes to the pathogenesis of many diseases including cancer and neurodegenerative disorders [104]. In particular, PARP inhibitors reduced cell death and microglial activation in RP models [105,106]. In our case, we observed colocalization of PAR accumulation in microglial cells at ONL. Raghuntha et al. argued that PARP was crucial to elicit the proinflammatory microglial response [107]. Evidence suggests that PARylation may promote macrophage/microglia polarization toward the M1 phenotype (proinflammatory) [108,109]. Maybe NUT reduced PARP overactivation and subsequent microglial activation (M1 phenotype) and cytokine production, or on the contrary. More studies are needed to clarify the relationship between PARP activity and microglial activation during the progression of RP.

Regarding nutritional supplementation for inherited retinopathies or other more prevalent retinal diseases, we have to take into account the bioavailability of the nutraceutical compounds or their active metabolites. Many factors could affect intestinal absorption, metabolism, blood clearance, or even interaction with other compounds. Some of them may not cross the blood–retina barriers and may not properly reach the retina. Intestinal absorption may be low (e.g., carotenoids such as lycopene). In addition, these compounds may be differently metabolized by rodents (the most used animal models) and humans. There are also differences in gut microbiome composition and intestinal permeability between rodents and humans. The compounds of our mixture NUT were previously used both in preclinical models and clinical trials. It is well known that lutein and zeaxanthin (fat-soluble compounds) accumulate in the macula of humans. They are transported from the gut to the retina, through the bloodstream via, mainly, high-density lipoproteins (HDLs) to form the macula pigment [110]. Fat or other carotenoids consumption increases or decreases their absorption, and their bioavailability is different depending on the source (e.g., eggs vs. vegetables) [111,112]. When they are administered as supplements, their bioavailability is affected by the materials, processes used for the encapsulation, and the form in which they are administered [113].

Vitamin B6, or pyridoxine, is a water-soluble vitamin. Their most active form is pyridoxal 5´-phosphate (PLP) that is converted to free vitamin B6 and absorbed by the small intestine. After its absorption, which depends on the pH and is a carrier-mediated transport, it is converted back to PLP. The presence of fiber slightly reduces their bioavailability by 5–10%, whereas the presence of pyridoxine glucoside strongly reduces it (by 75–80%). While folates are the natural form of vitamin B9 in food, folic acid is the synthetic form. Natural folates rapidly lose biochemical activity in foods (days or weeks). However, folic acid is highly stable (months or years) and resistant to chemical oxidation. Folic acid absorption is pH dependent and with a carrier-mediated transport. Once in the liver, folic acid is reduced to tetrahydrofolate and released into the blood [114,115]. The tetrahydrofolate is responsible for transporting single-carbon groups (e.g., the methyl group) for biosynthesis or modification of DNA/RNA, repair of DNA, synthesis of methionine from homocysteine, and other chemical reactions related to cellular metabolism [116].

Retinol acetate is the acetate ester of retinol (the natural form of vitamin A). It is commonly used in nutritional supplements and as a food additive. Dietary vitamin A is mainly absorbed in the proximal portion of the small intestine. Retinol esters as retinol acetate suffer hydrolysis by pancreatic lipase in the intestinal brush border. Once into the cells, retinol is re-esterified, incorporated into chylomicrons, and secreted in the lymph [117]. After entering the blood circulation, part of chylomicrons is hydrolyzed in plasma, and chylomicron remnants are taken up by tissues, mainly the liver (storage). Liver stores vitamin A as retinol bound to a specific carrier protein, retinol-binding protein (RBP). This retinol bound to RBP enters target tissues as retina where it is essential for the visual cycle [118].

Se, Zn, and Cu are trace elements important to maintain cell function. Se is semimetal and stable at normal temperatures. Se absorption occurs mainly in the duodenum and caecum by active transport through a sodium pump. Se absorption is decreased by lead, sulfur, calcium, or iron. Se is taken up by erythrocytes, reduced by glutathione reductase, and transported to the liver. Se is mainly transported in form of selenoprotein P. It is mainly stored in the liver and muscle as selenomethionine. It is excreted by urine [119].

Zn absorption occurs in the small intestine. Then, it is released into the portal blood mainly bound to albumin and distribute in the body. Zn homeostasis is predominantly regulated by its intestinal absorption and excretion to the intestinal lumen (feces). Zn absorption depends on the Zn of the diet and its intestinal bioavailability. The absorption is mediated by a carrier. Zn absorption is increased by animal protein and citrate. However, high levels of phytate inhibit its absorption [120].

Cu absorption primarily occurs in the upper small intestine. A small amount of Cu is stored in the body because most Cu is excreted in bile (feces) and a small amount by urine. Cu homeostasis is regulated by intestine absorption and released by the liver into bile. Cu absorption is increased by animal protein, citrate, and phosphate. However, high levels of dietary Zn, phytate, or cadmium inhibit its absorption [121].

Therefore, it is important to consider these characteristics when designing human studies. In our case, the formulation NUT has been designed for human use, and then we adapted it for a mouse study. On the other hand, present some advantages such as their oral administration or their no harmful effect when they are taken at the proper dosage and under medical supervision. Finally, we are aware of the partial effect of this formulation on retinal function and degeneration. Potentially, we would increase their effect by increasing the dose regimen to daily supplementation. Further studies are needed to confirm it.

## 5. Conclusions

RP is a genetic disease but inflammation and oxidative stress seem to play an important role in its progression. Therapeutic strategies targeting both inflammation and oxidative stress could help to preserve the vision for a longer time. Nutraceuticals are natural compounds with antioxidant and anti-inflammatory properties. In this study, we observed that nutraceutical supplementation ameliorated the function and the morphology of the retina in a mouse model of RP. The neuroprotective effect was accompanied by an improvement in the redox status and a reduction of the inflammatory process. Therefore, the use of this combination of nutraceuticals could be useful for future studies in humans.

## Figures and Tables

**Table 1:** Dietary composition of macronutrients, minerals, and vitamins in the standard chow diet.

Nutrients	Components	Composition
Macronutrients	Crude protein	14.3%
	Fat	4.0%
	Carbohydrate	48.0%
	Crude fiber	4.1%
	Neutral Detergent fiber	18.0%
	Ash	4.7%
Minerals	Calcium	0.7%
	Phosphorus	0.6%
	Sodium	0.1%
	Potassium	0.6%
	Chloride	0.3%
	Magnesium	0.2%
	Zinc	70 mg/kg
	Manganese	100 mg/kg
	Copper	15 mg/kg
	Iodine	6 mg/kg
	Iron	175 mg/kg
	Selenium	0.23 mg/kg
Vitamins	Vitamin A	6.0 IU/g
	Vitamin D3	0.6 IU/g
	Vitamin E	120 IU/kg
	Vitamin K3	20 mg/kg
	Vitamin B1	12 mg/kg
	Vitamin B2	6 mg/kg
	Niacin	54 mg/kg
	Vitamin B6	10 mg/kg
	Pantothenic acid	17 mg/kg
	Vitamin B12	0.03 mg/kg
	Biotin	0.26 mg/kg
	Folate (folic acid)	2 mg/kg
	Choline	1030 mg/kg

**Table 2:** Gene expression of inflammatory molecules in retinas from rd10 mice with or without oral administration of nutraceuticals.

Gene	C Relative Expression (Mean ± SEM)	rd10 Relative Expression (Mean ± SEM)	rd10 + NUT Relative Expression (Mean ± SEM)
TNFα	1.0 ± 0.0	40.8 ± 8.0 ***	5.8 ± 1.4 *
TNFR1	1.0 ± 0.1	2.3 ± 0.4 *	0.9 ± 0.2 ^bbb^
IL-6	1.0 ± 0.0	7.8 ± 2.0 **	2.5 ± 0.6 ^bb^
IL-1β	1.0 ± 0.0	17.0 ± 3.0 ***	3.6 ± 0.8 ^bb^
IL-18	1.0 ± 0.1	0.4 ± 0.1 *	0.1 ± 0.0 ***
GFAP	1.0 ± 0.0	8.3 ± 1.0 ***	2.6 ± 0.5 **^b^

Note: Gene expression (relative expression) in each group. Differences between untreated rd10 (rd10) mice and control (C) or NUT-treated rd10 mice (rd10 + NUT) were analyzed using Kruskal–Wallis test and post hoc Dunn´s multiple comparisons tests. C, control. * *p* < 0.05; ** *p* < 0.01; *** *p* < 0.001 for differences between control and rd10 mice; and ^b^ *p* < 0.05; ^bb^ *p* < 0.01; ^bbb^ *p* < 0.001 for differences between rd10 and rd10 + NUT mice.

## Data Availability

Data available in a publicly accessible repository.
